# USER CHALLENGES AND PREFERENCES FOR VOICE INTERFACE DESIGN ON SMART SPEAKERS AMONG LOW-INCOME MINORITY OLDER ADULTS

**DOI:** 10.1093/geroni/igae098.1904

**Published:** 2024-12-31

**Authors:** Jane Chung, Natalie Mansion, Madeleine Viceconte, Reiley Syros, Jodi Winship

**Affiliations:** Emory University School of Nursing, Atlanta, Georgia, United States; Virginia Commonwealth University, Richmond, Virginia, United States; Virginia Commonwealth University, Richmond, Virginia, United States; Virginia Commonwealth University, Richmond, Virginia, United States; Virginia Center on Aging, Virginia Commonwealth University, Richmond, Virginia, United States

## Abstract

Voice-assisted smart speakers offer benefits for independent living and wellness, particularly for individuals with mobility disabilities, vision impairments, or dexterity issues. Voice user interfaces (VUIs) integrated into smart speakers enable hands-free and eyes-free interaction, allowing users to perform tasks through voice commands. Despite increasing research on older adults’ adoption and interaction with voice-operated smart speakers, the specific usability challenges faced by low-income, minority older adults remain largely unexplored. We synthesized qualitative findings from two smart speaker-related projects, focusing on low-income, primarily African American older adults. Employing qualitative descriptive methods, including matrix analysis and content analysis, we explored usability issues and VUI design preferences. Overall, participants expressed positive experiences with hands-free interactions. However, some encountered difficulties due to a lack of instruction on using different buttons or specific commands to navigate to or exit certain functions. Users also experienced unintentional voice assistant activation, requiring careful command monitoring. While many participants were satisfied with the voice assistant’s gender, tone, and accent, occasional misunderstandings or actions that users do not comprehend have been noted. Participant interviews highlighted the significance of designing VUI that can be customized to accommodate users’ health conditions and speech abilities, given the prevalence of poorly managed chronic conditions and dental health issues among low-income minority older adults. This customization is essential to smart speaker adoption and use, as health outcomes such as stroke or missing teeth may affect users’ ability to pronounce certain words or articulate commands understood by the voice assistant.

